# Editorial: The impact of nicotine and e-cigarettes on mental health

**DOI:** 10.3389/fpsyt.2024.1446934

**Published:** 2024-06-27

**Authors:** Valeria Lallai, Kristine C. McGrath

**Affiliations:** ^1^ Neurobiology and Behavior Department, University of California, Irvine, Irvine, CA, United States; ^2^ School of Life Sciences, University of Technology Sydney, Sydney, NSW, Australia

**Keywords:** e-cigarette (e-cig), nicotine, cannabis, brain, public health

Tobacco and nicotine addiction continue to cast a looming shadow over public health, imposing a substantial burden on societies around the globe. Although progress has been made in reducing tobacco smoking rates in many developed nations, a new challenge has emerged on the horizon: the proliferation of e-cigarette use, particularly among demographics that were previously untouched by tobacco cigarettes. This alarming trend has ignited concerns about the potential health risks associated with e-cigarette use, an area where our understanding remains shrouded in uncertainty. In response to this evolving public health landscape, we present this editorial to delineate the scope and aspirations of our Research Topic, aiming to compile a comprehensive body of research that elucidates the current state of evidence on the use of e-cigarettes.

## Navigating the e-cigarette research landscape

To embark on a journey toward evidence-based e-cigarette research, it is imperative that we establish a cohesive framework for investigating this multifaceted issue. As we delve into the complexities of e-cigarettes, we must first address the approaches we employ in our quest for knowledge. Two studies published in this Research Topic canvased the use of e-cigarettes and nicotine in school settings. The first study (Leavens et al.), presented an overall concept and design of an ongoing trial that used the ECHO model to build capacity in schools to address youth e-cigarette use in Kansas. Pilot findings indicate that the ECHO model could lead to evidence-based policy changes with increased involvement from both students and staff in e-cigarette prevention and cessation. A second study (Affolter et al.) assessed the usage patterns of tobacco products, nicotine products (including e-cigarettes) and cannabis products among adolescents in Switzerland. Survey results indicated students who regularly used products were most likely to smoke cigarettes. These studies collectively provide valuable information for understanding and addressing the complex landscape of e-cigarette research, encompassing prevention strategies, prevalence data, and health implications.

## Unveiling the impact on the brain and peripheral organs

E-cigarettes, often touted as a safer alternative to traditional tobacco, have garnered significant attention due to their potential effects on the human brain and peripheral organs. What do we know so far about the effects of e-cigarettes on the brain and peripheral organs? Are there discernible alterations in neural function or damage to vital organs that can be attributed to e-cigarette use? Current evidence, including that of Vargas-Medrano et al., who explored genetic factors influencing nicotine addiction, suggests that more research is needed into how e-cigarettes may alter neural function or damage vital organs.

## Genetic modifications and long-term consequences

Beyond the immediate health effects, we must investigate the possibility that e-cigarettes cause long-term genetic modifications. Do these devices, with their assortment of chemical constituents, induce genetic alterations with lasting consequences? Research by Carreño and Lotfipour demonstrated that genetic variants, specifically in the alpha(α)6 nicotinic acetylcholine receptor subunit, may play a critical role in nicotine addiction behavior. Their study found that adolescent rats with a human CHRNA6 polymorphism exhibited enhanced nicotine-seeking behavior when exposed to nicotine cues. This suggests that genetic modifications induced by substances in e-cigarettes could similarly exacerbate addictive behavior, highlighting the urgent need to unravel the genetic impact of e-cigarette use to comprehend its full influence on human health.

## Charting the future of e-cigarette research

As we embark on this journey, we must also look to the horizon. What does the future hold for e-cigarette research? What new frontiers and challenges await us in the quest for knowledge? Research by Chen et al. highlighted that the prevalence of adolescent electronic nicotine use is rising, raising significant public health concerns due to its potential to progress into combustible cigarette smoking and to condition the developing brain for addiction to other drugs of abuse. Additionally, understanding the sex-specific genetic factors that influence nicotine addiction is essential as they may mediate substance use behaviors differently in men and women. By contemplating the future and continuing to investigate these genetic and behavioral dimensions, we can better position ourselves to address emerging issues and make informed decisions in the ever-evolving landscape of e-cigarette use.

## Exploring the nexus of e-cigarettes and drug intake behaviors

Another intriguing aspect of e-cigarette research is its potential connection to subsequent drug intake behavior. How do e-cigarettes influence or interact with the propensity to engage in drug consumption? A study by Smethells et al. highlighted that while nicotine may not be inherently more reinforcing in individuals with ADHD, other factors such as non-nicotine constituents and sensory experiences may play a significant role. This intersection warrants careful examination to understand the broader implications of e-cigarette use, especially considering the higher prevalence of smoking behavior in individuals with ADHD.

Additionally, a study by Gellner et al. shed light on the reinforcing effects of nicotine and other tobacco constituents, demonstrating that adolescents have higher rates of drug-seeking behavior compared to adults, and respond more intensely to stress and cue-induced reinstatement of nicotine-seeking behavior. This underscores the importance of addressing age-specific factors in the development of addiction and the implementation of cessation programs.

Moreover, Bautista et al. reviewed current evidence linking electronic nicotine cigarette use with potential health consequences, including an association between drug use and depression in humans. They explored the biological effects of individual constituents in electronic cigarette aerosols, such as propylene glycol, vegetable glycerin, nicotine, and flavorants, along with unlabeled ingredients like carbonyls and heavy metals. These findings highlight the need for further studies to understand the long-term clinical relevance of aerosol inhalation and its impact on drug intake behavior.

## Policy, regulatory frameworks, and demographic dynamics

Policy and regulatory frameworks play a pivotal role in shaping the patterns of e-cigarette usage among various age groups. What insights can research offer into the impact of regulations and legal constraints on e-cigarette usage? Understanding the interplay between policy and behavior is vital for crafting effective public health strategies. A study by Affolter et al. highlighted how weak policy regulations in Switzerland have led to higher prevalence rates of adolescent use of tobacco and nicotine products compared to other countries like Canada, the United States, and England. This finding underscores the need for stronger regulatory measures to curb e-cigarette usage among youth.

## Initiation and continuation factors

Finally, to combat the rise in e-cigarette use, we must unravel the complex web of factors associated with initiation and continuation. What drives individuals to take up e-cigarettes, and what compels them to persist in their usage? By identifying these factors, we can develop targeted interventions to reduce e-cigarette consumption. A randomized controlled trial by Schuster et al. examined the efficacy of varenicline combined with brief behavioral counseling and text message support for vaping cessation in adolescents. This study aims to assess biochemically verified continuous vaping abstinence and explore the impact of these interventions on reducing nicotine dependence in adolescents. Understanding the effectiveness of such interventions is crucial for developing comprehensive strategies to address e-cigarette use in this vulnerable population​.

We hope this Research Topic will provide the reader with a comprehensive and evidence-based understanding of e-cigarette use and its implications for public health. By delving into the questions outlined above and fostering collaboration among research groups, we aim to shed light on this pressing issue. Together, we can navigate the uncharted waters of e-cigarette research and contribute to the betterment of society’s health.

## Author contributions

VL: Writing – review & editing, Writing – original draft, Visualization, Validation, Supervision, Project administration, Conceptualization. KM: Writing – review & editing, Writing – original draft, Visualization, Validation, Supervision, Project administration, Conceptualization.

